# An Adapted Questionnaire Tailored for Assessing the Risk of Vitamin D Deficiency in Children That Is Proving Useful in Guiding Clinical Interventions

**DOI:** 10.3390/nu16070971

**Published:** 2024-03-27

**Authors:** Valeria Calcaterra, Hellas Cena, Rachele De Giuseppe, Ginevra Biino, Roberta Grazi, Matteo Manuelli, Sara Zanelli, Veronica Tagi, Alessandra Vincenti, Gianvincenzo Zuccotti, Valentina Fabiano

**Affiliations:** 1 Department of Internal Medicine and Therapeutics, University of Pavia, 27100 Pavia, Italy; 2 Pediatric Department, Buzzi Children’s Hospital, 20154 Milano, Italy; roberta.grazi@unimi.it (R.G.); zanelli.sara01@gmail.com (S.Z.); veronica.tagi@unimi.it (V.T.); gianvincenzo.zuccotti@unimi.it (G.Z.); valentina.fabiano@unimi.it (V.F.); 3 Laboratory of Dietetics and Clinical Nutrition, Department of Public Health, Experimental and Forensic Medicine, University of Pavia, 27100 Pavia, Italy; rachele.degiuseppe@unipv.it (R.D.G.); m.manuelli88@gmail.com (M.M.); alessandra.vincenti@unipv.it (A.V.); 4 Clinical Nutrition and Dietetics Service, Unit of Internal Medicine and Endocrinology, Clinical Scientific Institute Maugeri IRCCS, 27100 Pavia, Italy; 5 Institute of Molecular Genetics, National Research Council of Italy, 27100 Pavia, Italy; ginevra.biino@igm.cnr.it; 6 Department of Biomedical and Clinical Science, University of Milano, 20157 Milano, Italy

**Keywords:** vitamin D, deficiency, children, questionnaire, screening

## Abstract

Background: The identification of vitamin D (VitD) deficiency in pediatric populations is essential for preventive healthcare. We refined and tested the Evaluation of Deficiency Questionnaire (EVIDENCe-Q) for its utility in detecting VitD insufficiency among children. Patients and methods: We enrolled 201 pediatric patients (aged between 3 and 18 years). Clinical evaluation and serum vitamin D levels were assessed in all subjects. The EVIDENCe-Q was updated to incorporate factors influencing VitD biosynthesis, intake, assimilation, and metabolism, with scores spanning from 0 (optimal) to 36 (poor). Results: We established scores for severe deficiency (<10 mg/dL) at 20, deficiency (<20 mg/dL) at 22, and insufficiency (<30 mg/dL) at 28. A score of 20 or greater was determined as the optimal cut-off for distinguishing VitD deficient from sufficient statuses, as evidenced by ROC curve analysis AUC = 0.7066; SE = 0.0841; sensitivity 100%, 95% CI 0.561–1. The most accurate alignment was seen with VitD insufficiency, defined as 25-OH-D3 < 20 ng/mL. Conclusions: This study confirms that the EVIDENCe-Q is a valid instrument for assessing the risk of vitamin D deficiency and insufficiency in children. It offers a practical approach for determining the need for clinical intervention and dietary supplementation of VitD in the pediatric population.

## 1. Introduction

Vitamin D insufficiency and deficiency are widespread globally, with a significant prevalence observed [1]. In Europe, the overall occurrence of inadequate vitamin D intake is roughly estimated at 13%, and particularly in Italy, hypovitaminosis D affects over 50% of pediatric cases, especially during adolescent years [2,3].

As in adulthood, age factors impacting the acquisition, metabolism, or utilization of vitamin D pose risks for hypovitaminosis D during children [3]. These factors comprise limited natural sun exposure due to sunscreen use, dark skin pigmentation due to ethnicity, protective clothing, minimal sunlight exposure, and restricted outdoor time. Additionally, a dietary deficiency in foods rich in vitamin D, specific medications, obesity, and certain medical conditions affecting the absorption, utilization, or metabolism of vitamin D contribute to the risks associated with its deficiency [2,4,5,6].

For the enhancement of overall skeletal growth and health outcomes, understanding the levels of 25-hydroxyvitamin D in serum is crucial. Yet, due to logistical challenges associated with venipuncture and the financial constraints linked to laboratory assays for measuring blood vitamin D concentrations, direct blood testing at a population level is not feasible due to impracticality and high costs [6,7,8,9,10]. An alternative approach lies in leveraging the existing body of literature, which identifies various factors associated with serum or plasma vitamin D concentrations. These factors can be assessed through questionnaires to devise straightforward scoring systems aimed at identifying children at risk of hypovitaminosis [11,12].

In 2013–2014, Tran et al. [7], Sohl et al. [13], and Lopes et al. [8] demonstrated that risk scores evaluating demographic characteristics and lifestyle factors in old individuals can predict hypovitaminosis D; they showed the potential for creating a risk model for vitamin D deficiency in older individuals based on basic patient characteristics.

Similar correlations have been demonstrated by Bertrand et al. [10], Touvier et al. [14], and Deschasaux et al. [15] in adults; by Bjørn Jensen et al. [9] in pregnant women [14]; and by Nabak et al. [11] in postmenopausal women [15]. Moreover, a moderate correlation was found by Bolek-Berquist et al. [16] in young adults.

In 2021, Moschonis et al. [17], involving a cross-sectional epidemiological study of 2280 schoolchildren in Greece, demonstrated that the use of a scores evaluating age, sex, region, time exposition to screens, BMI, parental education, and season could predict vitamin D insufficiency and deficiency.

In the same year, De Giuseppe et al. [18] conducted the EVIDENCe-Q (Evaluation of Deficiency Questionnaire) study which introduced a novel questionnaire, designed to assess vitamin D deficiency in Italian adults. This questionnaire evaluated 20 items and attributed scores ranging from 0 to 36—scores directly related to deficiency. It has been demonstrated as a valuable screening tool for clinicians by accurately identifying individuals at risk of vitamin D deficiency; it helps clinicians to avoid unnecessary supplementation and blood tests, thereby streamlining clinical practice.

Inspired by the success of this initiative, our group conducted a study in 2022 to adapt and enhance the questionnaire for use in pediatric populations affected by obesity [2]. Researchers examined a revised rendition of the EVIDENCe-Q tailored for young people. Delving into a cohort of 120 pediatric patients, the questionnaire (comprising 20 multiple-choice queries) scrutinized various facets influencing the synthesis, assimilation, and intake of vitamin D and encompassing demographic, lifestyle and dietetic particulars, health status, pharmaceutical and supplemental regimens, sunlight exposure, and sunscreen application. The questionnaire’s precision was observed to be relatively lower in this demographic than in adults. These findings underscore the imperative for developing bespoke assessment tools catering to diverse and specific population subsets, such as infants, children, and adolescents. Contrary to the expectations, the score exhibited no discernible fluctuation corresponding to the severity of vitamin D deficiency, and no notable variance emerged concerning gender disparities or pubertal stages. Given the influence of adipose tissue, there was a compelling case for integrating additional elements into the scoring system, particularly for pediatric patients with obesity, while also taking into account potential metabolic complexities.

With its swift and user-friendly approach, the modified EVIDENCe-Q boasts enhanced efficacy. By scrutinizing supplementation details alongside lifestyle, dietary habits, and clinical nuances, it emerges as a robust tool for gauging vitamin D status. This makes it invaluable for periodic reassessment post-supplementation—typically advised after six months (barring exceptional cases)—and for promoting healthier habits among children and their families [2].

This study aimed to validate a modified pediatric questionnaire for assessing the risk of vitamin D deficiency and insufficiency. Following an adaptation process for a pediatric population, the revised questionnaire was administered to a new sample. The aim was to develop a scoring system with improved accuracy in identifying vitamin D deficiency, thereby rendering it an invaluable asset in clinical settings.

Given the challenges inherent in direct serum testing for vitamin D levels, research has pivoted towards non-invasive, cost-effective screening methods. The foundation of such methods is laid by the established correlation between specific lifestyle factors and serum vitamin D concentrations. Consequently, questionnaires that can quantitatively assess these factors offer a promising avenue for identifying at-risk populations without the need for invasive blood draws. Thus, it became paramount to devise a risk assessment tool tailored for children—one that accounts for the unique physiological and lifestyle variances in this demographic. Building on the groundwork laid by the EVIDENCe-Q for adults, our study sought to customize and calibrate the questionnaire for a pediatric population. This involved not only adapting the questions to be age-appropriate but also refining the scoring mechanism to reflect the distinctive vitamin D dynamics in children.

The crux of this investigation was to subject the modified EVIDENCe-Q to rigorous validation processes. We aimed to assess the questionnaire’s efficacy in the early detection of vitamin D insufficiency and deficiency among children. A robust validation would cement the questionnaire’s role as a preventive tool, helping to circumvent the adverse effects of vitamin D deficiency on bone health, growth, and overall wellbeing in pediatric populations.

Additionally, this study was designed to enhance the precision of the modified EVIDENCe-Q, aiming for a level of accuracy that would make it a mainstay in clinical assessments. Through this validation, we anticipated establishing a reliable, non-invasive risk assessment tool. Such a tool would empower healthcare professionals to identify at-risk children promptly and would facilitate the implementation of early interventions.

Incorporating the feedback from the initial pediatric pilot study, our research focused on refining the questionnaire’s parameters. We hypothesized that a more nuanced understanding of the interplay between obesity and vitamin D metabolism could lead to the identification of new predictive factors. These insights would, in turn, be used to update the scoring algorithm, aiming for a tool that is not only predictive but also educational, fostering awareness and prompting lifestyle modifications where necessary.

With the advent of a validated questionnaire specific to the pediatric demographic, the medical community would be equipped with a streamlined approach to vitamin D deficiency risk assessment. This would also align with contemporary shifts in healthcare towards preventive medicine and patient empowerment. A validated tool has the potential to significantly reduce healthcare costs by decreasing the reliance on laboratory assays and by facilitating early, targeted interventions that could preempt the progression of deficiency into clinically significant conditions.

## 2. Patients and Methods

### 2.1. Patients

We consecutively enrolled 201 pediatric patients (aged between 3 and 18 years) who were referred to the Vittore Buzzi Children’s Hospital by their general practitioner or primary care pediatrician for auxological evaluation. Children with history of vitamin D deficiency, rickets, congenital malformation, chronic disorders influencing nutritional status, severe anemia, and known genetic polymorphisms in the vitamin pathway were excluded from the sampling list. Children were referred between September 2023 and January 2024.

Clinical evaluation and serum vitamin D levels were assessed in all subjects. In all subjects, the questionnaire for assessing the risk of vitamin D deficiency and insufficiency was administered.

This study was conducted in accordance with the Helsinki Declaration of 1975, as revised in 2008. The institutional ethics committee approved the protocol (Ethics Committee Milano Area 1; Study Registration 2020/ST/234 Date 29 July 2020; Protocol No. 0030785 Date 12 July 2021). After receiving information about this study, all participants or their guardians provided written consent.

### 2.2. Methods

#### 2.2.1. Auxological Evaluation

Weight, height, and pubertal stage were measured in all participants, and their BMI values were calculated. Anthropometric measurements were conducted as previously described [19]. Pubertal stages were categorized as follows: Tanner 1 = pre-pubertal stage 1; Tanner 2–3 = mid-pubertal stage 2; Tanner 4–5 = late-pubertal stage 3 [20,21].

BMI was calculated as body weight (kilograms) divided by height (meters squared). BMI was converted into BMI z-scores using WHO reference values [22,23].

#### 2.2.2. Assessment of Vitamin D Level

For all children, we considered 25(OH)D (ng/mL) serum levels that were routinely measured using commercial kits (Alinity i 25-OH VitD reagent kit, Libertyville Township, IL, USA) on the ARCHITECT system. To assess the vitamin D status of each subject, we utilized the 25(OH)D cut-off values proposed by the Italian Pediatric Society and the Italian Society of Preventive and Social Pediatrics, categorizing severe deficiency, deficiency, insufficiency, and sufficiency as 25(OH)D levels of <10 ng/mL, <20 ng/mL, between 20 and 29 ng/mL, and >30 ng/mL, respectively [3].

#### 2.2.3. Questionnaire Design and Scoring

Since the pediatric version of the self-administered 20-item EVIDENCe-Q showed to have low performance, the questionnaire’s items were analyzed in order to improve its reliability. Item analysis revealed that three questions had a very low inter-item correlation so they were eliminated; the sun exposure question was modified adding frequency and time band; the sunscreen question was modified as well by adding SFP > 30; and the following four questions were added to the questionnaire: familial autoimmune diseases, nighttime wakening, hours of sleep, and exposure to smoking.

After this revision, the self-administered 20-item EVIDENCe-Q includes multiple choice questions (13 in dichotomous and 7 in polytomous mode) investigating factors affecting VitD levels, their production/intake, absorption, and metabolism (Table S1). In particular, it investigates (i) geo-graphical information on the place of residence (north, south, and central Italy as well as urban and peri-urban home areas); (ii) skin phototype (I–IV); (iii) exposure to sunlight for at least thirty minutes, specifying if during all the year or only in some of the seasons, and how many times a week and if during the 10:00 a.m–15:00 p.m. slot (yes; no); (iv) habitual use of sunscreen with a sun protection factor (SPF), specifying if the SPF ≥ 30 (yes; no; only during summer) and the frequency of use of sunscreen during sun exposure (one time; two times; three or more times); (v) consumption of dietary sources of VitD (daily consumption of at least one portion of whole milk and fortified foods; weekly consumption of at least three portions of fat fish; weekly consumption of at least two whole eggs; weekly consumption of at least two portions of dairy products); (vi) pathologies that interfere with the production and/or absorption of VitD (e.g., liver failure, renal failure, nephrotic syndrome, hyperparathyroidism, intestinal malabsorption including Chron’s disease, ulcerative colitis, celiac disease, cystic fibrosis, eating disorders) (yes; no); (vii) medications that may interfere with the nutrient bioavailability and or requirements (e.g., anticonvulsants, antipsychotics, glucocorticoids, immunosuppressive corticosteroids, anti-retroviral, weight loss drugs, cholesterol-lowering drugs, laxatives) (yes; no); (viii) use of multivitamin supplements or supplements containing VitD (yes; no); (ix) familial autoimmune diseases, osteoporosis, or other osteoarticular diseases; (x) sleeping hours (>8, 6–8, <6); (xi) nighttime wakening; (xii) TV/PC screen hours before going to bed; (xiii) exposure to smoking.

The pediatric EVIDENCe-Q score ranges from 0 (equivalent to the best VitD status) to 36 (equivalent to the worst one). In particular, responses assuming behavior that did not lead to a deficiency were assigned a score of zero, while those potentially leading to deficiency were assigned a score of 1 (if the response mode was dichotomous) or greater than 1, increasing by one unit for each answer that assumed a progressively worse behavior.

### 2.3. Statistical Analysis

Data were managed and analyzed using STATA software version 18 (College Station, TX, USA). Sample characteristics were analyzed by standard descriptive statistics, and *t*-test was used to compare mean values of quantitative variables. Correlation between 25(OH)D serum values and the questionnaire’s total score was computed. Analysis of variance (ANOVA) was performed to analyze the score mean value in the three classes of VitD status (deficiency, insufficiency, sufficiency). Analysis of the ROC (receiver operating characteristic) curve was used to identify the threshold values of the questionnaire score, which are useful to discriminate between the subjects who are at risk of deficiency or insufficiency and those who are not. Finally, prevalences of VitD status using the identified cut-offs were computed.

### 2.4. Ethical Considerations

Prior to the initiation of this study, all procedures were thoroughly reviewed and approved by the relevant institutional ethics committee. This study was conducted in accordance with the ethical principles of the Declaration of Helsinki. Informed consent was obtained from all participants and/or their guardians after a comprehensive explanation of this study’s purpose, procedures, potential risks, and benefits.

### 2.5. Study Limitations

While designing this study, we acknowledged certain limitations. This study’s findings would be representative of the specific demographic and geographic location of the sample. As such, caution should be exercised when generalizing the results to other populations. Moreover, the reliance on self-reported data for certain variables may introduce reporting bias, which was mitigated through subsequent validation with objective measurements.

## 3. Results

### 3.1. Demographic and Baseline Characteristics

The study sample comprised 201 subjects, including 87 males (43.3%) and 114 females (56.7%), ranging in age from 10 months to 17 years and 10 months. Table 1 provides a detailed breakdown of the demographic and baseline characteristics. Even if the differences are not statistically significant, males have higher mean values for all the studied variables with respect to females.

### 3.2. Vitamin D Status in the Pediatric Population

The analysis of 25(OH) VitD levels revealed distinct values between genders. The mean levels were 25.5 ± 10.7 ng/mL (median 23.8 ng/mL; range 4–72) in males and 24.3 ± 9.59 ng/mL in females (median 24 ng/mL; range 4–51) and were not significantly different (*t*-test *p*-value = 0.413), indicating a similar risk profile for vitamin D deficiency across genders.

### 3.3. Questionnaire Score Analysis

The assessment of questionnaire scores in relation to vitamin D status showed no significant gender differences in vitamin D dosages or questionnaire scores. The mean questionnaire score was 19.2 ± 3.9, with the distribution across different vitamin D status categories described in Table 2.

### 3.4. ROC Curve Analysis

**Figure 1:**
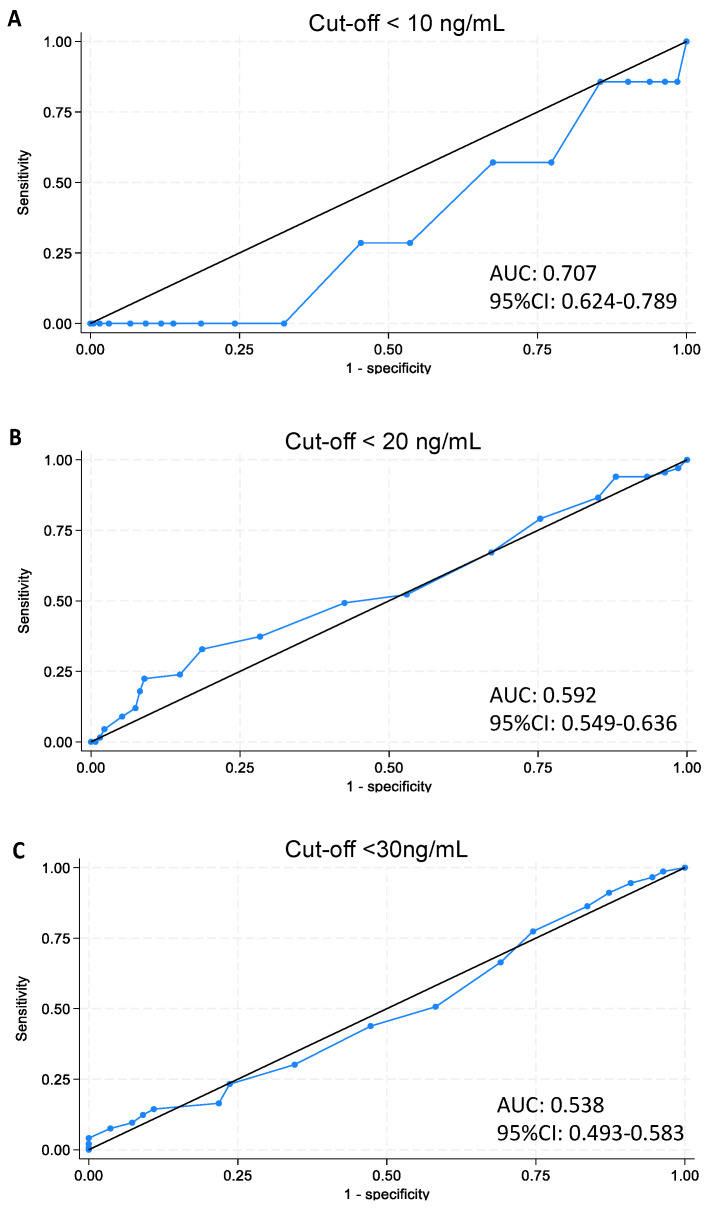
AUC for vitamin D deficiency. Panel (**A**): cut-off < 10 ng/mL. Panel (**B**): cut-off< 20 ng/mL. Panel (**C**): <30 ng/mL. Blue line = Receiver Operating Characteristic (ROC) curve.

The Receiver Operating Characteristic (ROC) curve analysis was conducted to identify optimal cut-off values for the questionnaire scores in predicting vitamin D deficiency and insufficiency. The Area Under the Curve (AUC) values from Figure 1A–C indicated varying degrees of accuracy in different deficiency classifications.

Optimal cut-off points, along with their sensitivity and specificity, are described in Table 3, highlighting the score’s effectiveness in differentiating between deficiency and sufficiency states. The analysis identifies the value ≥20 as the best threshold score on the questionnaire for discriminating between individuals deficient and non-deficient in vitamin D (ROC curve AUC = 0.7066; SE = 0.0841; sensitivity 100%, 95% CI 0.561–1.00).

### 3.5. Comparison of Vitamin D Deficiency and Insufficiency Prevalence

The comparison of vitamin D deficiency and insufficiency prevalence, based on the 25-OH-D3 levels and the questionnaire score cut-offs, is summarized in Table 4. This comparison revealed that the prevalence of vitamin D insufficiency is more closely aligned with the cut-off values derived from the ROC analysis for insufficiency defined as 25-OH-D3 < 20 ng/mL. It is noteworthy that these cut-off values, although defined for a pediatric population, show similarities to those established for adults.

### 3.6. Efficacy of the EVIDENCe-Q in Different Age Groups

Upon stratification of the cohort by age, distinct patterns emerged regarding the EVIDENCe-Q’s efficacy. The ability of the questionnaire to predict vitamin D deficiency was most pronounced in the 12–17 age group, with a questionnaire score mean of 20.3 ± 4.1, compared to 18.5 ± 3.7 in the 3–11 age group. While this trend suggests an increasing reliability with advancing age within the pediatric population, it is important to consider these findings in the context of the overall sample size and the statistical power of the study. Further research is necessary to validate the questionnaire’s predictive accuracy across age groups.

## 4. Discussion

In our comprehensive approach to the early detection of vitamin D deficiency in children, we have extended the scope of our previous study [18] to include a wider pediatric demographic. Our research underlines the critical role of vitamin D, obtained from both sunlight exposure and dietary intake, and the subsequent conversions to its active forms in the body, emphasizing the complexity of its bioavailability [2,3,4,24,25].

The adapted questionnaire, EVIDENCe-Q, was designed to encapsulate this complexity, integrating factors influencing both vitamin D2 and vitamin D3 levels. Vitamin D2, also known as ergocalciferol, is largely synthetized by the human body and is incorporated into food products. Conversely, vitamin D3, or cholecalciferol, is produced in human skin and is also obtained through the consumption of animal-based foods. In the liver, vitamin D2 and D3 undergo conversion to 25-hydroxyvitamin, also known as calcidiol, which is used as a biomarker for exposure [24,25]. Subsequently, it undergoes conversion in the kidneys into its most potent form, 1,25-dihydroxycholecalciferol or calcitriol. This enzymatic process occurs also in extrarenal sites, such as alveolar macrophages, osteoblasts, lymph nodes, placenta, colon, breasts, and keratinocytes, implying its potential autocrine/paracrine functions [25].

The widespread distribution of vitamin D receptors throughout the body points to the systemic repercussions of its deficiency [26,27,28]. Maintaining adequate vitamin D levels is essential for preventing a variety of health issues, making a compelling case for our non-invasive assessment tool in pediatric care.

Vitamin D action is mediated by a ubiquitous vitamin D receptor present in all nucleated cells. Given the widespread distribution of vitamin D receptors, which extend to the colon, osteoblasts, activated T and B lymphocytes and mononuclear cells, beta islet cells, brain, heart, skin, gonads, prostate, and breasts, it is anticipated that vitamin D deficiency will manifest in various extra-skeletal effects [26,27,28], such as an increased risk to the development of cardiovascular conditions, malignancies, immune system disorders, infections, chronic respiratory illnesses, and cognitive impairments [26,27,28,29,30,31]. For this reason, it is essential to promptly identify instances of vitamin D deficiency in order to mitigate these health concerns in children by preferably utilizing a non-invasive and cost-effective tool for their comfort.

The Italian Pediatric Society and Italian Society of Preventive and Social Pediatrics define a severe deficiency of serum 25-hydroxy concentration as below 10 ng/mL, define it as an inadequacy below 20 ng/mL, and as an insufficiency at 20–29 ng/mL. Concentrations of serum 25-hydroxy vitamin D exceeding 30 ng/mL are classified as satisfactory [3].

In line with our previous 2022 study [18], this research aimed to refine the sensitivity and specificity of the questionnaire, which is now inclusive of children across all BMI categories. The balanced gender distribution within our sample and the lack of significant differences in vitamin D levels between genders challenge the assumption that supplementation needs to differ significantly between male and female children.

The sample size, comprising 201 subjects, has shown insufficient power to establish statistically significant correlations between 25-OH-D3 levels and questionnaire scores when stratified by sex (43.3% males and 56.7% females) and age ranging from 10 months to 17 years with varying BMI values, sometimes undisclosed. This undermines the ability to draw definitive conclusions regarding the questionnaire’s diagnostic performance.

Moreover, the absence of expected differences in vitamin D levels across genders, along with non-significant Pearson’s correlation coefficients, necessitate a more conservative stance on the tool’s current effectiveness as an alternative to direct vitamin D measurements. Additionally, while a proven relationship between vitamin D and BMI exists, our investigation did not reveal a correlation that aligned with the extant literature.

The survey, comprising 20 multiple-choice questions, explored various factors that, according to the literature, could modify vitamin D levels included demographic details, dietary habits, medical history, medications, supplement usage, lifestyle choices, as well as exposure to smoking, sleep-related habits, and sunlight exposure, considering seasonal variations. Additionally, data on the use of sunscreen for sun protection were collected and analyzed. However, the consideration of seasonal variations has not consistently yielded the anticipated inverse relationship between vitamin D levels and questionnaire scores. This variation signals an area of potential enhancement for the tool’s sensitivity.

ROC curve analysis was intended to refine our tool’s predictive accuracy. However, the modest area under the ROC curve highlights the need for caution in interpreting these results due to the limited sample size. In light of these analyses, we still advocate that the EVIDENCe-Q remains a valuable educational tool by encouraging healthier behaviors, potentially impacting lifelong wellbeing; however, its utility for screening requires further substantiation.

We underscore the imperative for future research to involve larger, more diverse cohorts and longitudinal designs to robustly test the EVIDENCe-Q’s predictive validity and the impact of interventions informed by its findings.

Furthermore, the EVIDENCe-Q aims to identify at-risk populations without the necessity for invasive blood drawing; therefore, it does not include information about biological or biomolecular properties.

Future research endeavors should aim to expand the sample size, embrace longitudinal tracking of vitamin D levels and health outcomes, and evaluate the efficacy of interventions based on our findings.

## 5. Conclusions

Our study has significantly contributed to this field of knowledge by introducing a non-invasive method for assessing vitamin D status in children, offering a new avenue for the early detection of deficiencies. Acknowledging the limitations of our study, particularly in terms of the sample size, we recognize that our conclusions regarding the EVIDENCe-Q’s effectiveness in predicting vitamin D status require cautious interpretation.

This research makes a substantive contribution to the already vast body of knowledge, and the EVIDENCe-Q can be seen as a step towards simplifying the assessment of vitamin D status in diverse pediatric populations. However, until further studies are conducted, its use should be coupled with an awareness of its limitations.

Accurately establishing cut-off values is a critical goal. Clinicians may find that, until these cut-off values are validated by larger, more diverse studies, discerning which children might benefit most from supplementation and dietary modifications may not be straightforward. This nuanced understanding is essential for informed clinical decision making. Furthermore, while the potential for cross-gender applicability of cut-off values from the EVIDENCe-Q shows promise, it is clear that further research is needed to ensure these values are robust across various demographics. The aspiration for a simplified and effective assessment tool remains; however, it must be underscored that the current findings do not yet warrant a change in public health policy or clinical practice. Standardization of vitamin D status assessment should proceed cautiously, and any adjustments to public health policies and supplementation programs should await more conclusive evidence.

Our commitment to establishing a global standard for vitamin D assessment continues, with recognition of the need for widespread applicability and for rigorous validation. We are steadfastly progressing in our quest to devise a highly accurate, non-invasive screening tool that, in time, may serve as a reliable standard worldwide. In this light, our study’s endeavor is dual-purpose: it has yielded a pragmatic tool for immediate application and established a cornerstone for subsequent, more exhaustive research. The tailored EVIDENCe-Q for pediatrics not only heralds a potential shift in pediatric care practices but also exemplifies the dynamic and adaptable nature of patient-focused health care innovations.

## Figures and Tables

**Table 1:** Description of age and anthropometric data.

	Boys, *n* = 87			Girls, *n* = 114			*t*-Test
	Mean (SD)	Median	Min–Max	Mean (SD)	Median	Min–Max	*p*-Value
Age, years	9.1 (4.59)	9.67	0.8–16.9	9 (4.88)	9.6	1–17.9	0.873
Weight, kg	41.64 (25.94)	34.2	112.4–0	37.97 (24.27)	34.8	7.5–101.7	0.305
Height, m	1.34 (0.3)	1.38	1.8–0	1.29 (0.27)	1.35	0.7–1.8	0.195
BMI	21.1 (6.28)	19.92	36.7–0	20.99 (7.11)	19.91	9.5–47.4	0.918

**Table 2:** Questionnaire score by vitamin D status.

Vitamin D Status	N		Mean Score		Range	
	30 ng/mL Cut-Off	20 ng/mL Cut-Off	30 ng/mL Cut-Off	20 ng/mL Cut-Off	30 ng/mL Cut-Off	20 ng/mL Cut-Off
Deficiency	7	7	17 ± 3.11	17 ± 3.11	11–20	11–20
Insufficiency	139	60	19.4 ± 3.96	20.1 ± 4.29	11–30	11–29
Sufficiency	55	134	19.1 ± 3.89	19 ± 3.73	11–27	11–30

**Table 3:** Summary of ROC analysis results using the three reference variables.

	Deficiency 10	Insufficiency 20	Insufficiency 30
Optimal operating slope	1	1	1
Optimal cut-off	20	22	28
Optimal sensitivity	1	0.328	0.041
Optimal specificity	0.325	0.813	1
Clinical information statistic	0.325	0.142	0.041
Area under the ROC curve	0.707	0.592	0.538
SE of area (Hanley)	0.084	0.044	0.046
Sample size	201	201	201

**Table 4:** Prevalence comparison by vitamin D status and score cut-off identified by ROC curve analysis.

	25-OH-D3	Score Cut-Off
	*n* (%)	*n* (%)
Deficiency < 10 ng/mL	7/201 (3.48)	90/201 (44.78)
Insufficiency < 20 ng/mL	67/201 (33.33)	47/201 (23.38)
Insufficiency < 30 ng/mL	146/201 (72.64)	6/201 (2.99)

## Data Availability

All data are reported in this paper.

## Supplementary Materials

The following supporting information can be downloaded at: https://www.mdpi.com/article/10.3390/nu16070971/s1, Table S1: Pediatric EVIDENCe Questionnarie.

## References

[1] Cui A., Zhang T., Xiao P., Fan Z., Wang H., Zhuang Y. (2023). Global and regional prevalence of vitamin D deficiency in population-based studies from 2000 to 2022: A pooled analysis of 7.9 million participants. Front. Nutr..

[2] Calcaterra V., Cena H., Biino G., Grazi R., Bortoni G., Braschi V., Tomasinelli C.E., Schneider L., Zuccotti G. (2022). Screening Questionnaire for Vitamin D Insufficiency in Children with Obesity. Children.

[3] Saggese G., Vierucci F., Prodam F., Cardinale F., Cetin I., Chiappini E., De’ Angelis G.L., Massari M., Miraglia Del Giudice E., Miraglia Del Giudice M. (2018). Vitamin D in pediatric age: Consensus of the Italian Pediatric Society and the Italian Society of Preventive and Social Pediatrics, jointly with the Italian Federation of Pediatricians. Ital. J. Pediatr..

[4] Dominguez L.J., Farruggia M., Veronese N., Barbagallo M. (2021). Vitamin D Sources, Metabolism, and Deficiency: Available Compounds and Guidelines for Its Treatment. Metabolites.

[5] Song S., Yuan Y., Wu X., Zhang D., Qi Q., Wang H., Feng L. (2022). Additive effects of obesity and vitamin D insufficiency on all-cause and cause-specific mortality. Front. Nutr..

[6] Fradet C., McGrath P.J., Kay J., Adams S., Luke B. (1990). A prospective survey of reactions to blood tests by children and adolescents. Pain.

[7] Tran B., Armstrong B.K., McGeechan K., Ebeling P.R., English D.R., Kimlin M.G., Lucas R., van der Pols J.C., Venn A., Gebski V. (2013). Predicting vitamin D deficiency in older Australian adults. Clin. Endocrinol..

[8] Lopes J.B., Fernandes G.H., Takayama L., Figueiredo C.P., Pereira R.M. (2014). A predictive model of vitamin D insufficiency in older community people: From the São Paulo Aging & Health Study (SPAH). Maturitas.

[9] Bjørn Jensen C., Thorne-Lyman A.L., Vadgård Hansen L., Strøm M., Odgaard Nielsen N., Cohen A., Olsen S.F. (2013). Development and validation of a vitamin D status prediction model in Danish pregnant women: A study of the Danish National Birth Cohort. PLoS ONE.

[10] Bertrand K.A., Giovannucci E., Liu Y., Malspeis S., Eliassen A.H., Wu K., Holmes M.D., Laden F., Feskanich D. (2012). Determinants of plasma 25-hydroxyvitamin D and development of prediction models in three US cohorts. Br. J. Nutr..

[11] Nabak A.C., Johnson R.E., Keuler N.S., Hansen K.E. (2014). Can a questionnaire predict vitamin D status in postmenopausal women?. Public Health Nutr..

[12] Yamada C.D., Kuwabara A., Sakai Y., Okuno C., Mine A., Misaki S., Nishikawa T., Inoue N., Kishimoto N., Nishizaki Y. (2023). Usefulness of Vitamin D Deficiency Questionnaire for Japanese (VDDQ-J) for Screening of Vitamin D Deficiency and Low Muscle Mass in Relatively Healthy Japanese Anti-Aging Health Checkup Examinees. J. Nutr. Sci. Vitaminol..

[13] Sohl E., Heymans M.W., de Jongh R.T., den Heijer M., Visser M., Merlijn T., Lips P., van Schoor N.M. (2014). Prediction of vitamin D deficiency by simple patient characteristics. Am. J. Clin. Nutr..

[14] Touvier M., Deschasaux M., Montourcy M., Sutton A., Charnaux N., Kesse-Guyot E., Assmann K.E., Fezeu L., Latino-Martel P., Druesne-Pecollo N. (2015). Determinants of vitamin D status in Caucasian adults: Influence of sun exposure, dietary intake, sociodemographic, lifestyle, anthropometric, and genetic factors. J. Investig. Dermatol..

[15] Deschasaux M., Souberbielle J.C., Andreeva V.A., Sutton A., Charnaux N., Kesse-Guyot E., Latino-Martel P., Druesne-Pecollo N., Szabo de Edelenyi F., Galan P. (2016). Quick and Easy Screening for Vitamin D Insufficiency in Adults: A Scoring System to Be Implemented in Daily Clinical Practice. Medicine.

[16] Bolek-Berquist J., Elliott M.E., Gangnon R.E., Gemar D., Engelke J., Lawrence S.J., Hansen K.E. (2009). Use of a questionnaire to assess vitamin D status in young adults. Public Health Nutr..

[17] Moschonis G., Androutsos O., Hulshof T., Sarapis K., Dracopoulou M., Chrousos G.P., Manios Y. (2021). Risk evaluation of vitamin D insufficiency or deficiency in children using simple scores: The Healthy Growth Study. Nutr. Res..

[18] De Giuseppe R., Tomasinelli C.E., Cena H., Braschi V., Giampieri F., Preatoni G., Centofanti D., Princis M.P., Bartoletti E., Biino G. (2022). Development of a Short Questionnaire for the Screening for Vitamin D Deficiency in Italian Adults: The EVIDENCe-Q Project. Nutrients.

[19] Calcaterra V., De Giuseppe R., Biino G., Mantelli M., Marchini S., Bendotti G., Madè A., Avanzini M.A., Montalbano C., Cossellu G. (2017). Relation between circulating oxidized-LDL and metabolic syndrome in children with obesity: The role of hypertriglyceridemic waist phenotype. J. Pediatr. Endocrinol. Metab. JPEM.

[20] Marshall W.A., Tanner J.M. (1970). Variations in the pattern of pubertal changes in boys. Arch. Dis. Child..

[21] Marshall W.A., Tanner J.M. (1969). Variations in pattern of pubertal changes in girls. Arch. Dis. Child..

[22] Body Mass Inder-for-Age (BMI-for-Age). https://www.who.int/toolkits/child-growth-standards/standards/body-mass-index-for-age-bmi-for-age.

[23] BMI-for-Age (5–19 years). https://www.who.int/tools/growth-reference-data-for-5to19-years/indicators/bmi-for-age.

[24] Ross A.C., Taylor C.L., Yaktine A.L., Del Valle H.B., Institute of Medicine (US) Committee to Review Dietary Reference Intakes for Vitamin D and Calcium (2011). Dietary Reference Intakes for Calcium and Vitamin D.

[25] Zhang R., Naughton D.P. (2010). Vitamin D in health and disease: Current perspectives. Nutr. J..

[26] Marino R., Misra M. (2019). Extra-Skeletal Effects of Vitamin D. Nutrients.

[27] Lee J.Y., So T.Y., Thackray J. (2013). A review on vitamin d deficiency treatment in pediatric patients. J. Pediatr. Pharmacol. Ther. JPPT Off. J. PPAG.

[28] El-Sharkawy A., Malki A. (2020). Vitamin D Signaling in Inflammation and Cancer: Molecular Mechanisms and Therapeutic Implications. Molecules.

[29] Tarfeen N., Nisa K.U., Ahmad M.B., Waza A.A., Ganai B.A. (2023). Metabolic and Genetic Association of Vitamin D with Calcium Signaling and Insulin Resistance. Indian J. Clin. Biochem. IJCB.

[30] Carbone F., Liberale L., Libby P., Montecucco F. (2023). Vitamin D in atherosclerosis and cardiovascular events. Eur. Heart J..

[31] Sun J., Zhang Y.G. (2022). Vitamin D Receptor Influences Intestinal Barriers in Health and Disease. Cells.

