# Supplementary material for: An Adapted Questionnaire Tailored for Assessing the Risk of Vitamin D Deficiency in Children That Is Proving Useful in Guiding Clinical Interventions
Source: Nutrients. 2024 Mar 27;16(7):971. doi: 10.3390/nu16070971 (PMC11013822; doi:10.3390/nu16070971)
Supplement: Supplementary file 1 [file nutrients-16-00971-s001.zip › nutrients-2910695-supplementary.pdf]

Table S1: Pediatric EVIDENCe Questionnarie

____

DEVELOPMENT AND VALIDATION OF A QUESTIONNAIRE FOR THE IDENTIFICATION OF INDIVIDUALS AT RISK OF VITAMIN D DEFICIENCY
EVIDENCE-Q (Evaluation VItamin D dEficieNCy QUESTIONNAIRE)

Date (dd/mm/yyyy)

To be completed by your physician:

Date of birth/Age ______________ **Pubertal stage**______________ **Sex** M ☐ F ☐

[25-OH-D3] (ng/ml) ____________ on date ___________

Weight (Kg) ___________ Percentile______________
Height (m) ___________ Percentile______________
BMI (Kg/m^2) ____________ Percentile______________
Waist circumference (cm)___________
Ethnicity ______________

All questions are to be referred to your son/daughter:

1) In which area of Italy does your son/daughter live?
- North ☐
- Central ☐
- South ☐

2) Does your son/daughter live in an urban area?
- Yes ☐
- No ☐

3) In what skin phototype do you identify your son/daughter?
- Light - type I (always burns easily; never tan) ☐
- Light - type II (always burns easily; gets little tan) ☐
- Light - type III (always burns, mildly; gets a tan gradually) ☐
- Dark - type IV (gets burned minimally; always gets a tan, quickly) ☐
- Dark - type V (rarely gets burned; always gets a tan, quickly) ☐
- Dark - type VI (never gets burned; always gets a tan, quickly) ☐

4) Does your child consume at least 3 portions a week of fish, such as salmon, tuna, herring, trout, anchovies, swordfish, etc.?
- Yes ☐
- No ☐

5) Does he/she eat at least 2 eggs a week?
- Yes ☐
- No ☐

6) Does he/she consume at least one daily portion of whole milk or yogurt?
- Yes ☐
- No ☐

7) Does he/she consume at least 2 servings a week of dairy products such as cheese?

- Yes ☐
- No ☐

8) Does he/she consume at least one daily portion of products fortified with calcium and vitamin D (e.g., cereals, milk, yogurt)?

- Yes ☐
- No ☐

9) Is he/she taking vitamin D supplements or multivitamin supplements containing vitamin D?

- Yes ☐
- No ☐

If yes, which one? ____

How long has it been?

- Less than 3 months ☐
- More than 3 months but less than 6 months ☐
- More than 6 months but less than 9 months ☐
- More than 9 months ☐

10) Is your son/daughter affected by any of these conditions: liver failure, kidney failure, nephrotic syndrome, hyperparathyroidism, intestinal malabsorption (e.g., Chron's disease, ulcerative recto colitis, celiac disease, cystic fibrosis) or eating disorders?

- Yes ☐
- No ☐

11) Is your child taking any of the following drug therapies: anticonvulsants, antipsychotics, glucocorticoids, immunosuppressive corticosteroids, antiretrovirals, weight-loss drugs (e.g., Orlistat, Xenical, Ally), hypocolestrolemizers such as Ezetimibe, statins, bile acid sequestrants (cholestyramine), laxatives (long-term use)?

- Yes ☐
- No ☐

12) Does your child expose himself to sunlight for at least 30 minutes?

- Yes, all year round ☐
- Yes but only during the summer period ☐
- Yes but only during the winter period ☐
- Yes but only during the school term ☐
- No ☐

13) If you answered "yes" to the previous question, how often and in what time slots?

- ____ (specify number) times a week between 10:00 a.m. and 3:00 p.m.
- _____ (specify number) times a week in the other time slots

14) When your child is exposed to the sun, how often does he/she apply sunscreen?

- Never ☐
- Always ☐
- Only during summer, always or frequently ☐
- Only during summer, rarely ☐

15) When he/she applies sunscreen, does your child use one with a Sun Protection Factor (SPF) 30 or more?

- Yes ☐
- No ☐

16) Someone in your family has

- Autoimmune diseases ☐
- Osteoporosis ☐
- Other osteoarticular diseases ☐

17) How many hours does your son/daughter sleep?
- >8 hours ☐
- 6-8 hours ☐
- <6 hours ☐

18) Does your child have nocturnal awakenings and/or difficulty falling asleep and/or other problems during sleep?
- Yes ☐
- No ☐

19) How many hours does your child spend in front of the TV/PC screen before going to bed?
- Less than 1 hour ☐
- 1 to 3 hours ☐
- More than 3 hours ☐

20) Is your child exposed to smoke?
- Yes ☐
- No ☐
